# Surface Electromyography Meets Biomechanics: Correct Interpretation of sEMG-Signals in Neuro-Rehabilitation Needs Biomechanical Input

**DOI:** 10.3389/fneur.2020.603550

**Published:** 2020-12-03

**Authors:** Catherine Disselhorst-Klug, Sybele Williams

**Affiliations:** Department of Rehabilitation & Prevention Engineering, Institute of Applied Medical Engineering, Rheinisch-Westfälische Technische Hochschule (RWTH) Aachen University, Aachen, Germany

**Keywords:** surface electromyography, biomechanics, neuromechanics, clinical application, rehabilitation

## Abstract

Coordinated activation of muscles is the basis for human locomotion. Impaired muscular activation is related to poor movement performance and disability. To restore movement performance, information about the subject's individual muscular activation is of high relevance. Surface electromyography (sEMG) allows the pain-free assessment of muscular activation and many ready-to-use technologies are available. They enable the usage of sEMG measurements in several applications. However, due to the fact that in most rehabilitation applications dynamic conditions are analyzed, the correct interpretation of sEMG signals remains difficult which hinders the spread of sEMG in clinical applications. From biomechanics it is well-known that the sEMG signal depends on muscle fiber length, contraction velocity, contraction type and on the muscle's biomechanical moment. In non-isometric conditions these biomechanical factors have to be considered when analyzing sEMG signals. Additionally, the central nervous system control strategies used to activate synergistic and antagonistic muscles have to be taken into consideration. These central nervous system activation strategies are rarely known in physiology and are hard to manage in pathology. In this perspective report we discuss how the consideration of biomechanical factors leads to more reliable information extraction from sEMG signals and how the limitations of sEMG can be overcome in dynamic conditions. This is a prerequisite if the use of sEMG in rehabilitation applications is to extend. Examples will be given showing how the integration of biomechanical knowledge into the interpretation of sEMG helps to identify the central nervous system activation strategies involved and leads to relevant clinical information.

## Introduction

The coordinated activation of muscles forms the basis for human movement. A frequent consequence of lesions of the central nervous system is muscle paresis accompanied by reduced muscle force and/or the loss of the ability to activate the muscles in a coordinated way. This results in poor movement performance and causes pain and disability. To preserve and restore movement performance is a challenge, and the demand for more effective treatment methods is gaining more importance (1, 2). However, a more patient-tailored rehabilitation therapy would become possible, if the information about the subject's muscular activation is included in the treatment strategy (2, 3).

SEMG technologies allow the pain-free assessment of muscular activation and many ready-to-use technologies are available (4, 5). They enable sEMG measurements in several applications among which rehabilitation is of particular importance. Although sEMG could make an essential contribution to improved rehabilitation it has not yet been routinely translated to clinics (2). This crucial step can only occur, if sEMG achieves high acceptance by physicians and physiotherapists. However, the acceptance of sEMG by clinical users is currently low. Here, the technological challenge is to identify, adjust or develop sEMG tools, signal processing strategies and application procedures which enable sEMG to meet the users' expectations. sEMG can make an essential contribution to many clinical questions, but it also has many limitations. These limitations must be known, understood and integrated into analysis algorithms in order to enable a fast and correct interpretation of the sEMG signal (4, 6).

This perspective report is addressed to clinical users but especially to sEMG developers. The aim is to raise awareness of the importance of biomechanical factors in the analysis of sEMG signals acquired under non-isometric conditions.

### Barriers Limiting the Use of sEMG in Dynamic Conditions

One major barrier limiting the use of sEMG in clinical applications is that the correct interpretation of sEMG signals remains debatable (5, 7). Consequently, potential sEMG users will be in a predicament when applying sEMG technologies. The reason for this is that the number of factors influencing the sEMG signal are numerous and interwoven (4, 8). On the other hand, rehabilitative interventions are under increasing pressure to provide evidence of their impact. This is only possible if patient groups can be compared with each other or if individual patients can be matched to a baseline (2). This has been achieved in clinical routine through various tests and scores (9). Information about the amount of muscular activation during functional movement tasks is routinely not included in the assessment. There are various reasons for this and they are related to both educational and practical issues (2). One aspect among others is that in dynamic conditions many factors influence the sEMG signal that are difficult to control (4, 10). This often makes a comparison between subjects, muscles or contractions in dynamic conditions difficult and lowers the clinical potential of the information extracted from the sEMG signals (2).

The challenges sEMG is facing are related to the fact that most applications in rehabilitation are associated with non-isometric conditions. If information about the onset and cessation of muscular activation is to be extracted from the sEMG signal, unambiguous conclusions are possible as long as the sEMG signal can be related to individual movement phases. A particularly clinically relevant example for this approach is the determination of phases of muscular activation during gait (10). Thereby, onset and cessation of muscular activation are set in relation to the gait cycle intervals, which can be easily detected with foot switches. When the informative value is not the timing but the amount of muscular activation, it is necessary to rely somehow on the amplitude of the sEMG signal. However, sEMG amplitude as well as sEMG envelope are influenced by many different factors. Therefore, while the potential of sEMG amplitude is huge, information that can be obtained from it is rarely used in clinical applications. On the other hand, when the majority of factors affecting the sEMG signal are known, controlled and can be unambiguously determined, reliable conclusions can be drawn from the sEMG amplitude (4). This is the case in short duration applications involving isometric contraction. However, in non-isometric applications additional measurement methods are necessary to provide all relevant information needed (11, 12).

### Biomechanical Factors Influencing the sEMG Amplitude

The relationship between muscle force and sEMG signal has closely linked the disciplines of biomechanics and electromyography for decades (6, 13–17). Nevertheless, there is unfortunately no simple closed-form or equation that describes this relationship in an adequate manner. From biomechanics, it is well-known that the contraction force of the muscle fiber depends on the fiber length (4, 18–20), as well as on the contraction velocity (4, 13, 16). Both, the muscle fiber force-length relationship (21–23) as well as the muscle fiber force-velocity relationship (24–27) vary non-linearly. Considering in particular the force-velocity relation, it becomes clear that the force generated by a single muscle fiber is greater in eccentric contraction than in concentric contraction (28, 29). Therefore, there is also a dependency of the sEMG signal amplitude on the type of contraction (30–33). On a more macroscopic level, the torque generated by a muscle depends on its biomechanical moment (18, 34, 35), and thus on the joint position (36, 37). In isometric contractions, the moment arm of the muscle and center of rotation of the joint remain constant while in non-isometric applications both change resulting in an altered joint net torque and a modified muscle force (4).

These biomechanical factors affect the number of muscle fibers which must be excited to generate the force necessary to execute the movement. Since the sEMG amplitude depends on the number of excited muscle fibers, it is obvious that in non-isometric contractions sEMG amplitude varies with different biomechanical factors. In addition, agonistic, synergistic and antagonistic muscles generally act on a common joint and produce a resulting total net joint torque (15, 16, 37–41). Due to this redundancy of the musculoskeletal system, the central nervous system's activation strategies for different synergistic as well as antagonistic muscles have to be taken into consideration. These central nervous system control strategies are rarely appreciated in physiological movements and are hard to manage in pathology (42). Consequently, these complex and interrelated factors that underlie the relation between the sEMG amplitude and the force produced by both the muscle fibers as well as the entire muscle have to be taken into consideration when interpreting the sEMG signal in dynamic conditions. Table 1 summarizes on the effect of the five most relevant biomechanical factors that significantly influence the sEMG signal in non-isometric contractions and therefore require special consideration. All these factors are intrinsic and cannot be directly controlled (4). One possibility here is to learn as much as possible about the movement performed by the use of additional measurement methods and to integrate this knowledge into the analysis of the sEMG signal.

**Table 1:** Biomechanical factors that significantly influence the sEMG signal in non-isometric contractions.

	**Biomechanical factor**	**Effect**
1.	Muscle length	According to the force-length relation, the muscle fiber generates different forces at different sarcomere lengths. Sarcomere length changes with joint position.
2.	Contraction velocity of the muscle	The muscle fiber generates different forces at different contraction velocities due to the force-velocity relation. Contraction velocity is related to the angular velocity of the joint.
3.	Lever arm of the muscle	The angle at which the tendon attaches to the bone depends on the joint position. Since the resulting contraction force acts parallel to the tendon, the lever arm of the muscle varies with joint position.
4.	Type of contraction (concentric or eccentric)	The force-velocity relation is either increasing or decreasing depending on whether the muscle shortens or lengthens during contraction.
5.	Redundancy of the musculoskeletal system	Besides the agonist, antagonists and other synergistic muscles also contribute to the net joint torque.

Normalization of the sEMG amplitude to force or torque is frequently used to counteract the high variability of the sEMG signal (43). It allows the comparison between groups, subjects and conditions. However, especially in clinical application, there are reservations regarding amplitude normalization, since it can mask changes related to disease or therapy. This is an important aspect and normalization of the sEMG signal is, therefore, not always the method of choice when analyzing clinical sEMG data associated with abnormal or pathological cases.

## sEMG Meets Biomechanics and the Resulting Potential for Rehabilitation

Many disorders that require rehabilitation are associated with the altered control and activation of muscles by the central nervous system and with the progressive development of rheological modifications in soft tissues, joint deviations and deformities that alter the biomechanics of the system and add mechanical boundaries (44). Prominent examples are stroke, paraplegia or infantile cerebral palsy. Due to the complexity of the analysis of sEMG signals recorded in dynamic conditions, primitive muscle synergies have been successfully applied in recent years to differentiate between a pathologically altered muscular activation and a physiological control of the muscles by the central nervous system (45–48). The concept of muscle primitive synergies aims to reduce the complexity of motor control. The drawback of this reduction in complexity, however, is that it is often difficult to attribute specific changes in muscle synergies to individual symptoms such as spasticity, rigidity or compensatory patterns. This requires a more detailed analysis of the control strategy used by the central nervous system.

In order to be able to determine for each patient specifically, which individual alterations the activation strategy occur during the execution of movements, the physiological activation pattern of the muscles involved should be used as a baseline (49, 50). However, the amount of muscular activation and the resulting sEMG signals depend significantly on the biomechanical factors described in Table 1. A promising way to establish comparability and reproducibility between groups or different test sessions, when determining the amount of muscular activation, is to limit the analysis to near-isometric epochs (4). In terms of biomechanical factors, a near-isometric epoch means that only those sEMG signal segments are compared that are derived from the same contraction type as well as at similar muscle lengths, leverage conditions and contraction velocities.

Von Werder et al. referred to the separation of the sEMG signal into near-isometric epochs as categorization (51). They used the categorization approach to investigate the effect of movement velocity on the central nervous system's control strategies. Muscular activation of the elbow flexors and extensors was investigated during elbow flexion and extension tasks against a constant external torque over the full range of motion. Fifteen healthy subjects were included and movement tasks were performed with different self-selected movement velocities. sEMG was recorded from biceps, brachioradialis and triceps. By rectification and smoothing, the sEMG envelope was built and normalized to 75% of its maximal value. In addition, the elbow flexion and extension angle was determined using 3D motion analysis and the angular velocity was calculated by differentiation with respect to time. A total of 40 categories were formed, with each category being characterized by the three biomechanical factors: contraction type (concentric or eccentric), joint angle interval (25°-44°; 45°-64°; 65°-84°; 85°-104°, and 105°-125°) and angular velocity interval (30°/s-49°/s; 50°/s-69°/s; 70°/s-89°/s; 90°/s-110°/s). To identify near-isometric epochs in the sEMG signal, each sample of the normalized sEMG envelope was assigned to the category, which corresponds to the biomechanical situation at that point in time when the sample was taken. Afterwards, all sEMG envelope samples that belonged to a near-isometric category were averaged. Detailed description of the categorization approach can be found in Von Werder et al. (51) and von Werder and Disselhorst-Klug (42).

**Figure 1:**
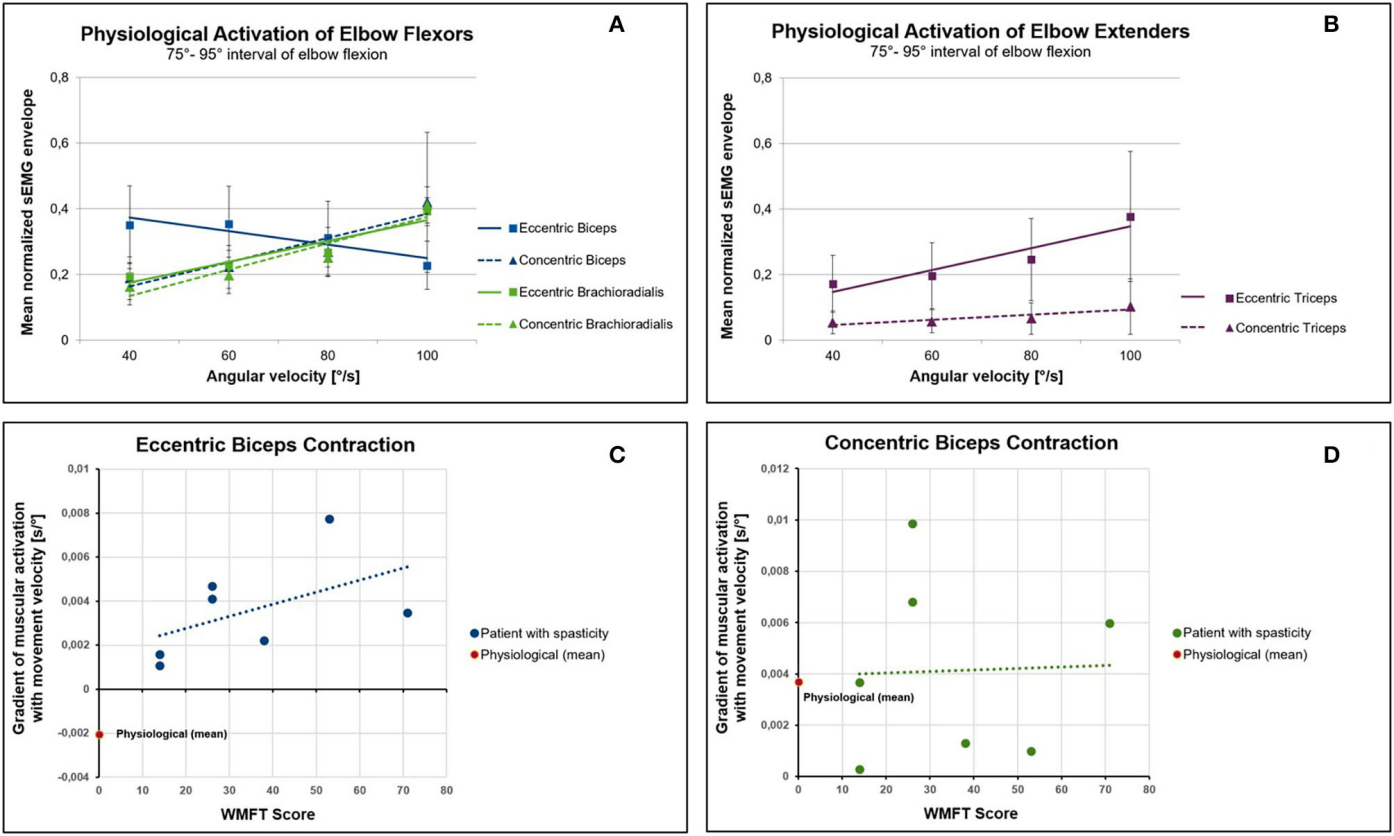
Effect of movement velocity on muscular activation in physiology and in patients suffering from spasticity. **(A)** Mean normalized sEMG envelope of the elbow flexors biceps and brachioradialis during concentric and eccentric contraction. In contrast to the biceps and to the force-velocity relationship, the muscular activation of the brachioradialis increases with increasing angular velocity. **(B)** The muscular activation of the triceps increases with increasing velocity of movement both in eccentric and concentric activation. **(C)** Relation between the severity of upper limb impairment (WMFT score) and the gradient of the normalized sEMG envelope with the movement velocity. A higher WMFT score means a more severe impairment related to spasticity. In patients with spasticity, the eccentric contraction of the biceps causes an increase in muscular activity with increasing movement velocity (positive gradient). This is in contrast to the physiological baseline, which is characterized by a negative gradient. **(D)** No differences could be found between patients and controls in concentric contractions.

Figures 1A,B show the effect of movement velocity on the sEMG envelope. In accordance with the force-velocity relation, the force that a single sarcomere can produce in concentric contraction decreases with increasing contraction velocity. Thus, if the external torque remains constant, concentric contraction requires more muscle fibers to be activated as the movement velocity increases. As a consequence, in concentric contraction the sEMG envelope increases with increasing movement velocity in all three muscles (Figures 1A,B).

In contrast to concentric contractions, the force that a single sarcomere can produce in eccentric contractions increases as the velocity of muscle stretch increases. Based on this biomechanical consideration, the sEMG envelope should decrease with increasing movement velocity during eccentric contractions. However, this can only be noticed in the biceps (Figure 1A). The sEMG signals of brachioradialis and triceps clearly show an increased envelope with increasing velocity in eccentric contractions (Figures 1A,B). These results can be better explained by control strategies via the central nervous system rather than by muscle biomechanics.

Particularly in rehabilitation, there is a great demand to be able to evaluate functionality in everyday situations (9). This is why the concept of including biomechanical knowledge in the analysis of the sEMG signals becomes more crucial for rehabilitation. A clinically relevant example, is spasticity. According to Lance spasticity is characterized by a velocity-dependent increase in tonic stretch reflexes (52) and does not include impaired voluntary movement and an abnormal posture (53). Although more recent publications differentiate the term spasticity (54), the definition introduced by Lance of the velocity dependence of the increase in the stretch reflex remains unchanged. Therefore, in the assessment of spasticity sEMG is commonly used to investigate the muscles' response to stretch (55–58). Although the investigation of muscles' response to stretch provides fundamental information about spasticity, it does not provide any indication of the occurrence or severity of spasticity during intentionally executed movement tasks relevant for patients' daily lives (55). In addition, muscular coordination is often investigated during gait analysis of patients suffering from disorders accompanied by spasticity (49, 59–65). In this case it is usually not possible to distinguish between spasticity and the voluntary compensatory activation needed to counteract for weakness. Although the assessment of spasticity is important for clinical management (54), it still lacks objective assessment methods to quantify the level of spasticity during intentionally executed movement tasks (9, 55, 58, 65–70).

Since, according to the definition of Lance, stretch velocity dependency is a characteristic property of spasticity, the question arises whether the integration of biomechanical knowledge might support a distinction between voluntary muscle activation and spasticity. As discussed before, to achieve comparability between physiological and spastic muscular activation patterns in dynamic conditions, near-isometric epochs of the sEMG signal have to be compared. Figures 1C,D compare the gradient of the normalized sEMG envelope against the movement velocity of healthy subjects with that obtained for 7 patients suffering from spasticity of the biceps muscle with different degree of severity. Study design and sEMG post processing were identical to the procedure described above. The gradient of the sEMG envelope with angular velocity was calculated and averaged over all joint angle intervals. This was done for each patient separately. Upper limb motor ability was clinically assessed using the Wolf Motor Function Test (WMFT).

As in healthy subjects, the biceps' sEMG envelope increases in patients with increasing contraction velocity during concentric contractions. This relationship is reflected by a positive gradient (Figure 1D). However, this is in contrast to eccentric contractions (Figure 1C). While healthy subjects show a negative gradient in eccentric contractions (Figure 1A), patients show an increase in the amount of muscular activation with increasing movement velocity. This positive gradient can neither be explained by the force-velocity relation nor does it correspond to a physiological central nervous system's control strategy. Thus, a positive gradient of the muscular activation with movement velocity in eccentric phases of muscular contraction can be interpreted as a sign of spasticity. When the magnitude of the gradient is compared to the WMFT score (Figure 1C), it becomes apparent that a more severe spasticity (higher WMFT score) tends to be associated with a higher gradient.

## Discussion: Bringing sEMG Technologies to Clinical Use

The sEMG signal is significantly dependent on various biomechanical factors and it can be assumed that an interpretation of the sEMG signal with respect to amplitude becomes more accurate when these biomechanical factors are taken into account. Hence, isometric measurements, in which these factors can be controlled, are still widely used. In rehabilitation, however, isometric contractions usually play a subordinate role. Here, the analysis of intentionally executed movement tasks is of primary importance. This complicates the interpretation of the sEMG signals, if beside the timing, the magnitude of the muscular activation is of interest. The consequences are often contradictory results both between individual studies and between follow-up measurements. This is fatal for the translation of the sEMG into clinical application, as it essentially weakens the users' confidence in the methodology.

Among others, Bogey et al. emphasized the clinical relevance of an integrated analysis of timing and relative magnitude of the sEMG signal (10). Consequently, new ways have to be found to increase the reliability the information gained from the magnitude of sEMG signal in dynamic conditions. The consideration of at least the essential biomechanical factors could lead to an improved informative value of the sEMG signals. This leads to two consequences:

1. When analyzing non-isometric conditions, additional measurement methods must be applied synchronously to the sEMG signal, which provide information about the execution of movements, such as movement cycle intervals, joint positions, movement velocities and external forces. This approach is already successfully applied in clinical gait analysis (71) and needs to be extended to other scenarios.
2. On the basis of current biomechanical knowledge, information about the execution of the movement must be merged with the sEMG signal. This will probably only be reliable if the analysis is broken down into near-isometric epochs. Appropriate algorithms that make this possible must be developed in the future.

The two examples given show how the consequent implementation of this strategy leads to new insights important for rehabilitation. They give a representative of the potential of integrating biomechanical principles into the interpretation of the sEMG.

In conclusion, there is an increasing demand in rehabilitation for objective methods, which on one hand provide evidence and on the other hand enable a therapy tailored more to the individual patient. sEMG has the potential to contribute significantly to this goal, even in dynamic conditions (2). However, biomechanical factors should be more integrated in the analysis of sEMG signals in the future. This becomes more urgent when sEMG signals are recorded in dynamic conditions. New and innovative sEMG processing and information extraction strategies are needed to make this approach clinically applicable. These challenges cannot be solved by isolated research labs. A multi- and inter-disciplinary network is needed, which will collectively work toward the development and establishment of sEMG procedures to meet the demands and acceptance of physicians and therapists.

## Data Availability Statement

The data analyzed in this study is subject to the following licenses/restrictions: The copyright for the data belongs to the Department of Rehabilitation and Prevention Engineering; Institute of Applied Medical Engineering; RWTH Aachen. Requests to access these datasets should be directed to Catherine Disselhorst-Klug, disselhorst-klug@ame.rwth-aachen.de.

## Ethics Statement

The studies involving human participants were reviewed and approved by Ethik-Kommission der Medizinischen Fakultät an der RWTH Aachen. The patients/participants provided their written informed consent to participate in this study.

## Author Contributions

CD-K elaborated the relationship between the surface EMG and the biomechanical factors, identified the barriers for translation into clinical application, developed the measures necessary to overcome them, reinterpreted the data from the presented studies, and wrote the manuscript. SW conducted an extensive literature research on the context of the manuscript, involved in the reinterpretation of the study data, assigned the results to the state of the art, and revised the manuscript. All authors contributed to the article and approved the submitted version.

## Conflict of Interest

The authors declare that the research was conducted in the absence of any commercial or financial relationships that could be construed as a potential conflict of interest.
